# Robust Foreground Detection: A Fusion of Masked Grey World, Probabilistic Gradient Information and Extended Conditional Random Field Approach

**DOI:** 10.3390/s120505623

**Published:** 2012-05-02

**Authors:** Mohd Asyraf Zulkifley, Bill Moran, David Rawlinson

**Affiliations:** 1 Department of Electrical, Electronic and Systems Engineering, Faculty of Engineering and Built Environment, Universiti Kebangsaan Malaysia, Bangi 43600, Malaysia; 2 Department of Electrical and Electronic Engineering, The University of Melbourne, Melbourne, VIC 3010, Australia; E-Mails: wmoran@unimelb.edu.au (B.M.); davidjr@unimelb.edu.au (D.R.)

**Keywords:** foreground detection, shadow removal, Gaussian modelling, colour co-occurrence, conditional random field, edge-based modelling, colour constancy

## Abstract

Foreground detection has been used extensively in many applications such as people counting, traffic monitoring and face recognition. However, most of the existing detectors can only work under limited conditions. This happens because of the inability of the detector to distinguish foreground and background pixels, especially in complex situations. Our aim is to improve the robustness of foreground detection under sudden and gradual illumination change, colour similarity issue, moving background and shadow noise. Since it is hard to achieve robustness using a single model, we have combined several methods into an integrated system. The masked grey world algorithm is introduced to handle sudden illumination change. Colour co-occurrence modelling is then fused with the probabilistic edge-based background modelling. Colour co-occurrence modelling is good in filtering moving background and robust to gradual illumination change, while an edge-based modelling is used for solving a colour similarity problem. Finally, an extended conditional random field approach is used to filter out shadow and afterimage noise. Simulation results show that our algorithm performs better compared to the existing methods, which makes it suitable for higher-level applications.

## 1. Introduction

Foreground detection algorithms have been implemented in many applications such as people counting, face recognition, license plate detection, crowd monitoring and robotic vision. The accuracy of those applications is heavily dependent on the effectiveness of the foreground detection algorithm used. For example, some people counting systems will not work well when the surrounding illumination is low, such as during rainy days or inside dark rooms. Such a system will not be able to give a correct count because of the inability of the algorithm to distinguish between foreground and background objects. It is very important for the background modelling algorithm to be robust to a variety of complex situations. However, it is almost impossible to make such a system robust to all situations and conditions such as low variation in illumination change, reasonable movement speed and high contrast between background and foreground object. In fact, a majority of previous papers such as [1–3] only function well within limited conditions and constraints. Any slight deviation from the required conditions significantly degrades performance. Algorithms such as face recognition fail to perform properly once the constraints are violated. The aims of our work are to improve accuracy and robustness of background modelling to (1) sudden as well as gradual illumination change; (2) small movements of background objects; (3) colour similarity between foreground and background; and (4) shadow and afterimage noise. This paper is part of Zulkifley's [4] PhD thesis.

Illumination change is one of the key issues when robust video analytics are developed. The issue can be divided into the subcategories of local and global on one hand, while sudden and gradual on the other. Learning capability can be incorporated into background modelling to enable the algorithm to adapt to the surrounding change either instantaneously or gradually. However, to find a single good model that fits both slow and fast learning rate is a difficult task and too dependent on the situation. An example of algorithm developed for gradual illumination change is by Jimenez-Hernandez [5]. His works used independent component analysis by utilizing spatio-temporal data to classify the foreground and background pixels. Our approach to cope with sudden/gradual illumination change as well as the problem of small movements of background objects is to fuse good background modelling with a colour constancy algorithm. By using colour co-occurrence based background modelling [1], we are able to achieve good foreground detection even under moving background noise and gradual illumination change. The background learning constant is set to a slow rate for handling gradual illumination change. Prior to this, the colour constancy approach is used to transform each input frame into a frame as seen by a canonical illuminant. This step allows the algorithm to be robust to sudden illumination change. We improve the grey world algorithm [6] by introducing adaptive mask and statistical grey constants. We also modify the method by Renno *et al.* [7] to filter out noise due to variation in grey constant values modelled by a Gaussian distribution.

Other flaws in the method of [7] are the degradation in its performance both under low ambient illumination and where there is colour similarity between background and foreground. We exploit higher level information such as gradient and edge to solve these problems. However, we argue that gradient information alone is not enough to provide robustness to the system. We propose a method which fuses both gradient and intensity information for better detection. The colour co-occurrence method will provide the intensity aspect while improved edge-based background modelling by using a fattening algorithm and temporal difference frame edge will provide the gradient aspect. A Gaussian distribution is used to realize the probabilistic edge-based background modelling. Both intensity and gradient methods are combined before final filter is applied to remove noise, especially shadows. A Conditional random field (CRF) approach is used to remove shadow and afterimage probabilistically. The algorithm of Wang [3] is improved by using a new shadow model and by incorporating previous neighbourhood values for decision making. As a result, algorithms that depend on foreground detection will produce sharper foreground which contributes to overall accuracy improvement.

This paper is organized into 9 sections. A literature review will be explained in Section 2. Section 3 will discuss a brief overview of the system. The details of the algorithms will be explained in Sections 4–7. Then, simulation results and discussion are presented in Section 8. Finally, conclusions are drawn in Section 9.

## 2. Literature Review

The most cited work for background modelling is the mixture of Gaussian (MoG) approach introduced in 1999 by Stauffer and Grimson [2]. The method has proven to be effective in handling gradual illumination change for indoor and outdoor situations, but it still lacks in terms of robustness, especially for the problems of sudden illumination changes, moving background objects, low ambient illumination and shadows. Lee and Chung [8] then combined MoG with weighted subtraction method for health care surveillance system. Another method by Varcheie *et al.* [9] also implemented MoG through a region-based updating by using colour histogram, texture information and successive division of candidate patch. Instead of using a mixture of Gaussian distributions, Ridder *et al.* [10] predict and smooth out the mode of the pixel value by using Kalman filter. This algorithm suffers the same problem as both methods only use temporal information for their decision making. In [11], Wang *et al.* used alpha-stable distribution instead of Gaussian distribution to detect background clutter. Synthetic aperture radar is used to detect the presence of a ship, and they obtained less spiky image or reduced fluctuation in the image due to improved modelling. They found that the ship detection is less spiky based on synthetic aperture radar image. In order to reduce intensity fluctuations due to noise, Bozzoli *et al.* [12] and Yu *et al.* [13] applied intensity gradient in their background modelling. Their approaches were found to be good in suppressing intensity value fluctuations but tend to produce wrong detection when the background object is moving, as in the case of an escalator or shaking tree.

The most popular method of gathering statistical information for each pixel is to use a colour histogram approach as in [14,15]. Li *et al.* [16] introduced the colour co-occurrence method, invoking the relationship between two pixels in consecutive frames for background modelling. Their approach uses Bayes rule for classifying each pixel as either moving foreground or moving background. This approach performs well in handling gradual illumination changes and moving background noise. However, the image obtained is not crisp and the method failed under sudden illumination changes. Crispness of the image is the quality of the object boundary, whether the edge is clear or blurred. In 2005, Zhao and Tao [17] used a colour correlogram which relates two pixel values within a certain distance inside the same frame. The algorithm performs well as the input for tracking non-deformed objects. The weakness of this approach is that it cannot handle non-rigid objects, especially in human detection algorithms where human foreground shapes change continuously as they walk. Another two popular methods of gathering statistical information are the fuzzy histogram approach [18] and colour ratio histogram approach [19].

Robust foreground detection is hard to achieve if each pixel is treated separately from its neighbours and from corresponding pixels in preceding frames. In order to improve the accuracy of background modelling, more information should be incorporated for decision making. Instead of making decisions based on a single pixel value; spatio-temporal information is used for better detection. Each of these approaches is further classified into deterministic or probabilistic. Temporal information is obtained by including previous data in the determination of the current pixel value or label. Haritaoglu *et al.* [20] is one of the first papers to apply deterministic temporal information. The paper constructs the background model by using minimum and maximum intensity values, and the maximum intensity difference between consecutive frames during the training period. Some examples of probabilistic approaches can be found in the papers by Li *et al.* [16], Bozzoli *et al.* [12] and Barandiaran *et al.* [21]. Deterministic approaches normally employ fixed thresholds for decision making. Spatial information is important as it correlates each pixel with its neighbours. Spatial techniques assume that any pixel will have higher probability to be a foreground if the majority of its neighbours are foreground. The algorithm by Hsu *et al.* [22] is an example of a deterministic spatial information approach, while Kumar and Hebert [23] and Paragios and Ramesh [24] implement a probabilistic approach of background modelling using Markov random fields. Spatio-temporal methods combine both spatial and temporal information, and most such algorithms are more robust to complex situations. Deterministic spatio-temporal approaches such as the algorithm of Zhao *et al.* [25] achieve good foreground detection even during the night, and that of Pless [26] is suitable for robust outdoor surveillance applications. Examples of probabilistic spatio-temporal approaches that provide effective foreground detection are the work of Kamijo *et al.* [27] and Wang *et al.* [28]. Both algorithms use Markov random fields to model spatio-temporal information. In 2007, Wang [3] introduced CRF in background modelling to classify each pixel into foreground, background or cast shadow. This approach provides sharper foreground detection, especially for a scene that contains a lot of cast shadow noise.

Few works [29,30] have implemented colour constancy approaches to adapt their algorithms to illumination changes. Most of the existing colour constancy algorithms are built for image processing applications and will not perform well for video analytics applications. This is due to the complexity of video scenes, which poses a tough challenge to estimate the reflectance dynamics in consecutive frames. As the scenes evolve, the estimated reflectance will also vary. Thus fixed reflectance values in image processing are no longer accurate. The most popular colour constancy method is the grey world algorithm, which was introduced by Buchsbaum [6] in 1980. Since then, the algorithm has evolved rapidly into several forms. However, the main idea remains the same, namely to estimate the illuminant by using average intensity values. The major weakness of the original grey world algorithm is that it cannot distinguish moving objects for grey constant calculation. In [31], Finlayson *et al.* applied the grey world algorithm to comprehensive image normalization, whereby two images with different illuminants are transformed into their canonical form. Their algorithm iterates until it reaches a stable state. In 2003, Ebner [32] combined the white patch retinex and grey world approaches for producing the canonical image. Reflectances are obtained by applying both approaches in parallel. Local space average colour and maximum deviation are used to find the required adjustment. Renno *et al.* [7] have implemented the grey world assumption for video processing to both indoor and outdoor situations. Their algorithm performs well if the moving object in the scene is considerably small compared to the frame size. If the moving object is relatively large, it occupies most of the frame, which leads to bad grey constant values because moving pixels are used to determine the values.

## 3. Overview of the System

**Figure 1.**
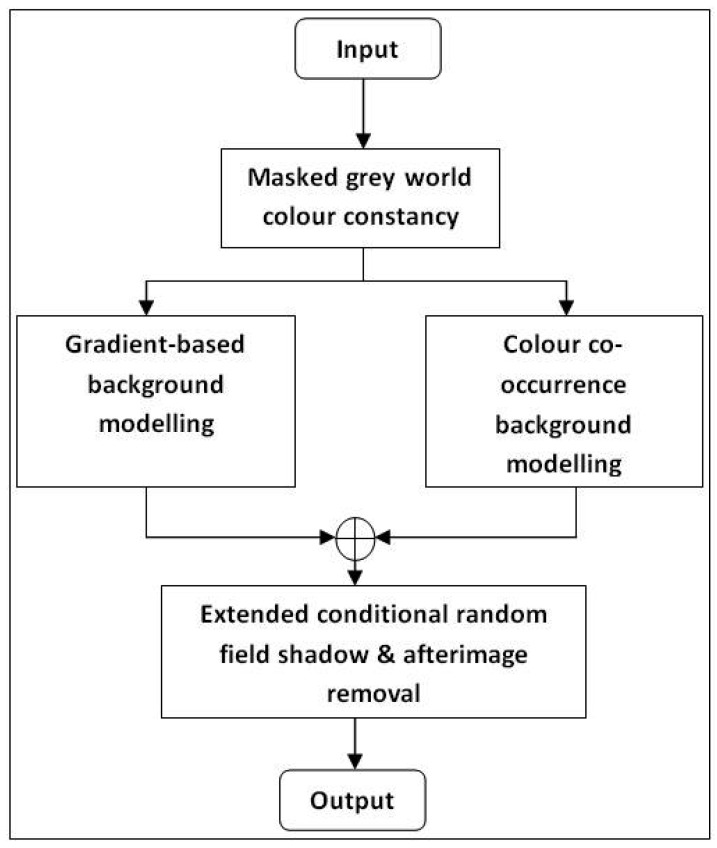
Block diagram of the overall system.

The goals of our algorithm are to improve background accuracy and modelling robustness to (1) sudden and slow illumination changes; (2) colour similarity between foreground and background objects; (3) shadows and afterimages; and (4) moving background objects. First, all input frames are transformed into canonical frames to solve the sudden illumination change problem. We then apply the grey world assumption and modify the algorithm by Renno *et al.* [7] to handle for more complex situations. The algorithm is improved by introducing a 2-stage mask and a probabilistic approach to determine grey parameter values used to exclude moving objects from inclusion in grey parameter calculations. Then, probabilistic gradient-based background modelling is fused with the colour co-occurrence algorithm by Li *et al.* [1]. Gradient information is used to address the problem of colour similarity between foreground and background objects. A combination of temporal difference frame edge and current input frame edge is found to be effective in distinguishing colour similarity. A colour co-occurrence approach also handles the problem of gradual illumination change and movement of background objects. Finally, shadow and afterimage removal is performed to obtain sharper foreground objects. The method by Wang [3] is improved by introducing a new shadow model, which is applied to a extended CRF model for decision making. This removal algorithm is applied only to pixels with label equal to 1 prior to the test. An overview of the whole system is shown in Figure 1.

## 4. Masked Grey World Colour Constancy

**Figure 2.**
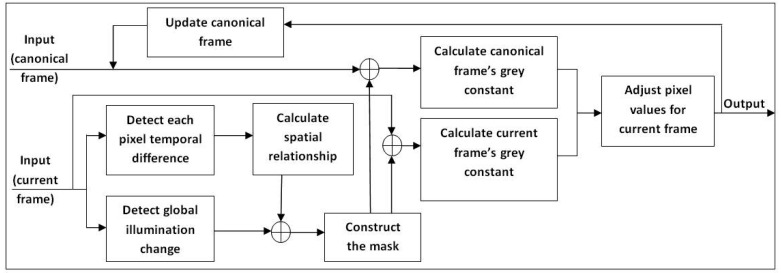
Block diagram of the masked grey world colour constancy algorithm.

This method was first introduced in [4,33] by Zulkifley and Moran. The aim of this section is to transform each frame into a canonical frame using the masked grey world algorithm. The motivation for applying colour constancy approach is to overcome sudden illumination change issue. Learning rate for background modelling can be set to lower value, which is good for handling slow change in the scene as any sudden change will be handle by colour constancy approach. The grey world algorithm assumes that the spatial average of surface reflectance in a scene is achromatic. Therefore, it is constant if there is no illumination change. This is true for outdoor environments where strong global illumination from sunlight will make other sources of light insignificant. However, the single average assumption is inaccurate for indoor environments, which usually have multiple sources of illumination. Previous grey world algorithms such as [6,31,32] are built for image processing applications, which assume no object movement between images. Some adjustments and alterations are required for video processing implementations because of the increased complexity of video content. In consequence, a 2-stage masking was introduced to improve the transformation accuracy of the algorithm by Renno *et al.* [7]. Figure 2 shows the simplified block diagram of the masked grey world algorithm.

The mask Mp is introduced to filter out moving objects from the grey world parameter calculation since the foreground object has no significant role in parameter calculation. This is done so that only stagnant pixels from both the reference frame *F_c_* and the current input frame will be used in calculating the grey world parameters. Since grey world algorithm take the average values of the pixels in the frame, moving object is an inaccurate representation of the grey constant value. By removing the moving foreground, better estimation of grey constant can be obtained, which leads to better colour adjustment. In addition, the mask also plays a role in detecting overcrowded scenes. For this, we use the whole frame to achieve better average values when the normal grey world algorithm fails to adjust appropriately to a scene overcrowded with foreground objects. We propose using a mask to filter out moving object pixels as well as to detect global illumination changes. The reason foreground pixels are not included in the grey constant calculation is that the canonical frame does not contain their information. The first frame is designated as the canonical frame *F_c_* and becomes the reference frame for the grey world parameter calculation. The first stage of calculating the mask involves classification of each pixel as belonging to a moving object or not. A Gaussian distribution is used to model the probability distribution of the temporal difference between the input frame, *F^t,x,y^*(*R,G,B*) and the preceding frame, *F^t^*^−1^*^,x,y^* (*R, G, B*). Variances $\left(\sigma_{R}^{2},\sigma_{G}^{2},\sigma_{B}^{2}\right)$ are assumed to be identical for all colour channels of RGB space. This assumption is applied throughout this chapter. Let *x* and *y* be the spatial coordinates of a pixel at a time instant *t*.

(1) $$P_{1}(F^{t,x,y},F^{t-1,x,y})∼NP(F_{j}^{t,x,y};F_{j}^{t-1,x,y},\sigma_{1}^{2}),\;j\in \left\{R,G,B\right\}$$

Then, the label of each pixel is obtained by comparing the temporal differences with a threshold value, *Τ*_1_, to classify it into a moving object or background pixel. The assigned label, $L_{1}^{t,x,y}$ of each pixel is set high if it belongs to a foreground object and low otherwise.

(2) $$L_{1}^{t,x,y}=\{\begin{array}{cc} 0 & \text{if}\;P_{1}(F^{t,x,y},F^{t-1,x,y})\geq {Τ}_{1}\\ 1 & \text{if}\;P_{1}(F^{t,x,y},F^{t-1,x,y})<{Τ}_{1} \end{array}$$

**Figure 3.**
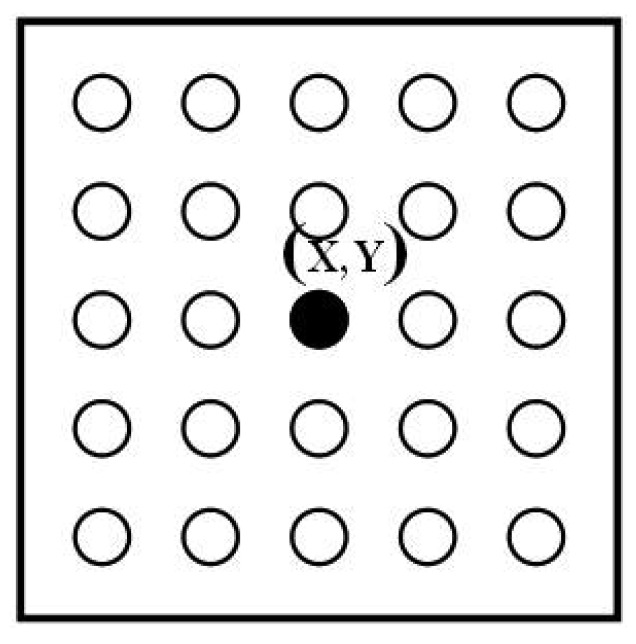
Example of 5 × 5 neighbourhood kernel of Kr_1_, Kr_2_, Kr_3_ and Kr_4_.

In order to filter out noise, the spatial correlation (*S^t,x,y^*) of each pixel and its neighbouring labels are used to determine the mask. A *k* × *l* kernel size is applied as the pixel neighbourhoods (Kr_1_) as shown in Figure 3.

(3) $$S^{t,x,y}={Τ}_{1}L_{1}^{t,x,y}+\sum_{\forall (x,y)\in \text{K}{\text{r}}_{1}}(1-{Τ}_{1})L_{1}^{t,x,y}$$

This is then compared with a threshold value *Τ*_2_ for assignment of the final label of the first-stage mask. Each pixel label remains high if a majority of the neighbouring labels are high and low otherwise. This is based on the assumption that a moving pixel should belong to a connected foreground region.

(4) $$Mp_{1}^{t,x,y}=\{\begin{array}{cc} 1 & \text{if}\;S^{t,x,y}>{Τ}_{2}\\ 0 & \text{if}\;S^{t,x,y}\leq {Τ}_{2} \end{array}$$

Once the initial mask is obtained, hypothesis testing based on the Neyman–Pearson method is performed to detect global illumination changes. If a change is detected, the mask (Mp_2_) will be the whole frame, which means every pixel will be considered in the grey world parameters calculation. This step is important in solving the problem of very crowded scenes with many moving objects. Usually, the first-stage mask will consist of only a small number of pixels, and this may lead to a wrong grey parameter value. For each pixel, the null hypothesis (*H*_0_) is modelled by the same Gaussian distribution as in Equation (1), while an alternative hypothesis (*H*_1_) is modelled as in Equation (6).

(5) $$\begin{array}{cc} P_{2}(H_{0}^{t,x,y})∼NP(F_{j}^{t,x,y};F_{j}^{t-1,x,y},\sigma_{2}^{2}), & j\in \{R,G,B\} \end{array}$$

(6) $$P_{2}(H_{1}^{t,x,y})=1-P_{2}(H_{0}^{t,x,y})$$

Detected changes in global illumination will be represented by the alternative hypothesis while the null hypothesis will represent no global illumination change. Both probabilities $P_{2}\left(H_{0}^{t,x,y}\right)$ and $P_{2}\left(H_{1}^{t,x,y}\right)$ are multiplied throughout the whole frame for finding the frame's $P_{2}\left(H_{0}^{t}\right)$ and $P_{2}\left(H_{1}^{t}\right)$.

(7) $$P_{2}(H_{0}^{t})=\prod_{\forall (x,y)\in F}P_{2}(H_{0}^{t,x,y})$$

(8) $$P_{2}(H_{1}^{t})=\prod_{\forall (x,y)\in F}P_{2}(H_{1}^{t,x,y})$$

Then, hypothesis testing based on Neyman–Pearson is performed to get the final mask. The null hypothesis will be rejected if Equation (9) is true.

(9) $$P_{2}(H_{1}^{t})>\eta_{1}P_{2}(H_{0}^{t})$$

Using the resulting mask, the grey world parameters Gc(R,G,B) are calculated for both the current input frame and the canonical frame. For every colour channel, the grey parameter is the intensity averaged over the masked pixels. Each channel is treated separately, so each channel has its own grey parameter values and let *T_n_* denote the total number of masked pixels.

(10) $$\begin{array}{ll} \text{Gc}(R,G,B)=\frac{\sum_{\forall (x,y)}F^{t,x,y}(R,G,B)}{\text{Tn}}, & (x,y)\in {\text{Mp}}_{2}^{t,x,y}=1 \end{array}$$

The colour adjustment ratio, Ar(R,G,B) between the grey world parameters of the canonical frame and the input frame is the ratio by which each colour channel will be scaled. In order to guarantee that the difference between the grey parameters of the current input frame and the canonical frame is not due to noise, the difference between those frames is modelled as a Gaussian distribution as in Equation (11). If the likelihood of the difference P_3_ is less than the threshold value (*Τ*_3_), the colour adjustment ratio is reset to 1; if it is not, the original ratio will be retained.

(11) $$\begin{array}{cc} P_{3}^{x,y}∼NP({\text{Gc}}^{t}F_{j}^{t,x,y};{\text{Gc}}^{t}F_{j,c}^{x,y},\sigma_{3}^{2}), & j\in \{R,G,B\} \end{array}$$

(12) $${\text{Ar}}^{t,x,y}(R,G,B)=\{\begin{array}{cc} {\text{Gc}}^{t} & \text{if}\;P_{3}^{x,y}>{Τ}_{3}\\ 1 & \text{if}\;P_{3}^{x,y}\leq {Τ}_{3} \end{array}$$

Final output of the masked grey world algorithm $F_{o1}^{t,x,y}(\text{R},\text{G},\text{B})$ is obtained by taking the dot product of the original input frame and colour adjustment ratio. Then the adjusted image is passed to both colour co-occurrence and probabilistic edge-based background modelling.

(13) $$F_{o1}^{t,x,y}(R,G,B)=F^{t,x,y}(R,G,B).{\text{Ar}}^{t,x,y}(R,G,B)$$

### Maintenance of Canonical Frame

It is important for the canonical frame to be updated continuously because of the “noise” between frames. An example of the “noise” is when the canonical frame was first captured with some parts blurred. The frame will be updated with a better value when later frames contain less blurred image. A fixed canonical frame gives wrong grey constant values when a background object leaves the scene, for example when an object is removed from the scene. The canonical frame is maintained using an infinite impulse response filter where *Τ*_4_ is a small positive value. Only masked pixels will be updated. *Τ*_4_ should be given a larger value to increase the pace of learning if the scene contains many moving background objects.

(14) $$F_{c}^{x,y}(R,G,B)=(1-{Τ}_{4})F_{c}^{x,y}(R,G,B)+{Τ}_{4}F_{o1}^{t,x,y}(R,G,B)$$

## 5. Review of Colour Co-Occurrence Background Modelling

**Figure 4.**
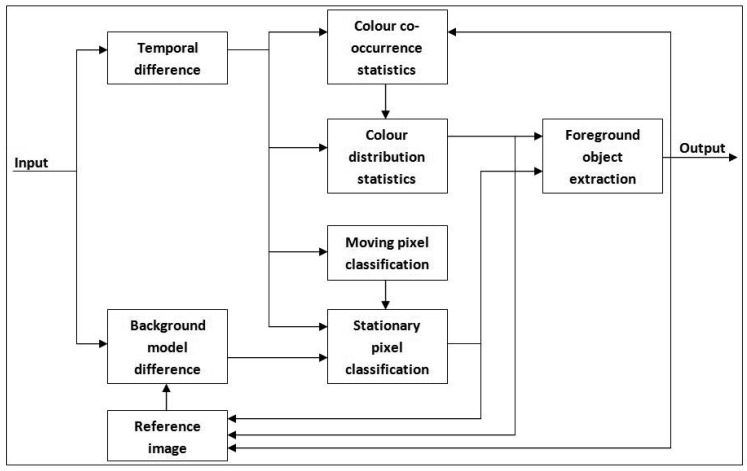
Block diagram of colour co-occurrence background modelling.

As shown in Figure 1, the transformed frame is fed to both colour co-occurrence modelling [1] and our edge-based foreground detection [34]. Both methods run concurrently so that they can compensate for each other's weaknesses. The reason for choosing the colour co-occurrence algorithm as the basis for foreground detection is its ability to distinguish between moving background and moving foreground pixels. A detailed explanation of the original algorithm can be found in [1] and [16]. The algorithm utilizes inter-frame colour co-occurrence as the input to a Bayesian decision rule so that each moving pixel can be classified either as moving background *bc* or moving foreground *fc*. Block diagram of the subsystem is shown in Figure 4.

Simple background subtraction is used to find both moving foreground and moving background pixels. Colour co-occurrence statistics are applied to filter out the moving background. Let $F_{o2}^{t,x,y}$ be the output of colour co-occurrence algorithm. Initial background frame $\left(F_{\text{bd}}^{t,x,y}\right)$ is obtained by using frame subtraction with respect to $F_{r}^{t,x,y}$. A global threshold is applied to classify the pixel either as a moving object or static object. Using a similar method, a temporal difference frame $\left(F_{\text{td}}^{t,x,y}\right)$ is obtained by frame subtraction between $F_{o1}^{t,x,y}(R,G,B)$ and $F_{o1}^{t-1,x,y}(R,G,B)$. For each pixel where $F_{\text{td}}^{t,x,y}$ is bigger than zero, a colour co-occurrence (*c^t^, c^t^*^−1^) pair is extracted, which is then compared with the values stored in the table of colour co-occurrence statistics, $S_{\text{cc}}^{t,x,y}$.

(15) $$S_{\text{cc}}^{t,x,y}:=\{\begin{array}{l} p_{\text{cc}}^{t,x,y}=p(c^{t},c^{t-1}|x,y)\\ p_{\text{ccb}}^{t,x,y}=p(c^{t},c^{t-1}|\text{bc},x,y)\\ c^{j,x,y}=(R^{j,x,y},G^{j,x,y},B^{j,x,y}),\text{where}\;j=0\;\text{or}\;1 \end{array}$$

A Bayesian decision approach is used to classify which probabilities of background change (*P_bc_*) and foreground change (*P_fc_*) are modelled as follows

(16) $$P(\text{bc}|c^{t},c^{t-1},x,y)=\frac{P(c^{t},c^{t-1}|\text{bc},x,y)P(\text{bc}|x,y)}{P(c^{t},c^{t-1}|x,y)}$$

(17) $$P(\text{fc}|c^{t},c^{t-1},x,y)=\frac{P(c^{t},c^{t-1}|\text{fc},x,y)P(\text{fc}|x,y)}{P(c^{t},c^{t-1}|x,y)}$$

Moving background is recognized if the probability of background change is bigger than the probability of foreground change:

(18) $$P(\text{bc}|c^{t},c^{t-1,x,y})>P(\text{fc}|c^{t},c^{t-1,x,y})$$

The universal set of colour co-occurrence changes between the frames can only be caused either by moving foreground or moving background.

(19) $$P(c^{t},c^{t-1}|x,y)=P(c^{t},c^{t-1}|\text{bc},x,y)p(\text{bc}|x,y)+P(c^{t},c^{t-1}|\text{fc},x,y)P(\text{fc}|x,y)$$

The decision rule will be further simplified by substituting Equations (16)–(18) into Equation (19):

(20) $$2P(c^{t},c^{t-1}|\text{bc},x,y)P(\text{bc}|x,y)>P(c^{t},c^{t-1}|x,y)$$

Both *P*(*c^t^, c^t^*^−1^|*x,y*) and *P*(*c^t^, c^t^*^−1^|*bc, x,y*) are obtained from the table of colour co-occurrence statistics while *P*(*bc*|*x,y*) is extracted from $F_{o1}^{t,x,y}(R,G,B)$. If $F_{\text{td}}^{t,x,y}$ is bigger than zero, the colour co-occurrence (*c^t^, c^t^*^−1^) of that pixel is extracted, which will be compared with the stored statistical values. If a match is found, the corresponding probabilities are retrieved and inserted into Equation (20) for detecting moving background. If no match is found, both probabilities are assumed to be zero. The labelling for temporal inter frame change $\left(F_{\text{td}}^{c,t,x,y}\right)$ is classified as in Equation (21).

(21) $$F_{\text{td}}^{c,t,x,y}=\{\begin{array}{ll} 0 & \text{if}\;F_{\text{td}}^{t,x,y}=0\\ 1 & \text{if moving background}\\ 2 & \text{if moving foreground} \end{array}$$

The final label for both backgrounds and temporal differencing are as follows

(22) $$F_{\text{bd}}^{\text{fc},t,x,y}=\{\begin{array}{ll} 0 & \text{if}\;F_{\text{bd}}^{t,x,y}=0\;\text{or}\;F_{\text{td}}^{c,t,x,y}=1\\ 1 & \text{otherwise} \end{array}$$

(23) $$F_{\text{td}}^{\text{fc},t,x,y}=\{\begin{array}{ll} 0 & \text{if}\;F_{\text{td}}^{c,t,x,y}\leq 1\\ 1 & \text{if}\;F_{\text{td}}^{c,t,x,y}=2 \end{array}$$

The output frame for colour co-occurrence algorithm is obtained by using a pixel-wise **OR** operator between $F_{\text{bd}}^{\text{fc},t,x}$ and $F_{\text{td}}^{\text{fc},t,x,y}$. Finally, **OPEN** and **CLOSE** operators are performed to clean up the output.

(24) $$F_{o2}^{t,x}=F_{\text{bd}}^{\text{fc},t,x,y}\vee F_{\text{td}}^{\text{fc},t,x,y}$$

## 6. Probabilistic Edge-Based Background Modelling

**Figure 5.**
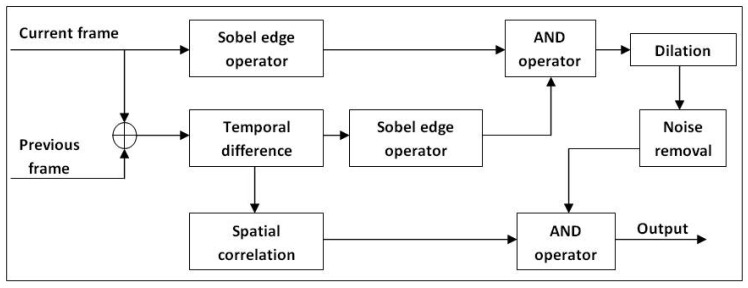
Block diagram of probabilistic edge-based background modelling.

Our probabilistic edge-based background modelling is constructed primarily to deal with the colour similarity issue between background and foreground objects. The method proposed by Li *et al.* [1] alone is not sufficient to produce good detection in the case of colour similarity because many foreground pixels are miscategorized as background pixels. We approach this problem by exploring higher-level information, especially edges. Edge information is known to be more robust to illumination change [35], leading us to explore the effect of manipulating moving edges. The basis of our edge-based background modelling is the fusion between the temporal frame's edge $\left(F_{\text{tde}}^{t,x,y}\right)$ and the current frame's edge $\left(F_{\text{ie}}^{t,x,y}\right)$. Figure 5 shows the framework of the proposed subsystem.

All edge detections are performed based on the Sobel edge operator [36]. The temporal difference frame (*F_td_*) that relates the current frame to previous frame is modelled by Gaussian distribution. The acquired probability *P*_4_(*F^t,x,y^, F^t^*^−1^*^,x,y^*) is then checked against a threshold value *Τ*_5_. The pixel is set to high if the corresponding probability is bigger than *Τ*_5_ and vice versa.

(25) $$\begin{array}{ll} P_{4}(F^{t,x,y},F^{t-1,x,y})\sim NP\left(F_{j}^{t,x,y};F_{j}^{t-1,x,y},\sigma_{4}^{2}\right), & j\in \{R,G,B\} \end{array}$$

(26) $$F_{\text{tde}}^{t,x,y}=\{\begin{array}{cc} 1 & \text{if}\;P_{4}(F^{t,x,y},F^{t-1,x,y})>{Τ}_{5}\\ 0 & \text{if}\;P_{4}(F^{t,x,y},F^{t-1,x,y})\leq {Τ}_{5} \end{array}$$

After that, the temporal frame edge and current input frame edge are extracted. Both edge frames are then fed into an AND operator to remove noise and afterimage. Let ${\text{L}}_{2}^{t,x,y}$ be the binary map which will be set high if both $F_{\text{tde}}^{t,x,y}$ and $F_{\text{ie}}^{t,x,y}$ are high.

(27) $${\text{L}}_{2}^{t,x,y}=\{\begin{array}{ll} 1 & \text{If}\;F_{\text{tde}}^{t,x,y}=F_{\text{ie}}^{t,x,y}=1\\ 0 & \text{otherwise} \end{array}$$

Since the output obtained from ${\text{L}}_{2}^{t,x,y}$ will only add fine lines to the foreground detection, dilation is applied to increase the detection accuracy. The additional noise from the dilation process will be filtered out by Equation (31). Thus, the additional noise is kept at the minimum. This step proved to be critical in increasing detection accuracy for situations where foreground and background colour are similar. Dilation is performed using the decision rule given by Equation (28) where the size of the neighbourhood kernel Kr_2_ is *k* × *l* pixel.

(28) $${\text{L}}_{3}^{t,x,y}=\{\begin{array}{ll} 1 & \text{if any}\;{\text{L}}_{2}^{t,x,y}=1,\forall (x,y)\in {\text{Kr}}_{2}\\ 0 & \text{otherwise} \end{array}$$

From the temporal difference frame in Equation (27), spatial correlation is added to smooth out the noise. Later, it will be convolved with *u* × *u* kernel (Kr_3_) before being compared with a threshold value, *Τ*_6_. Let (*a_i_, a_j_*) be the kernel anchor. The sum of all kernel elements should be equal to one.

(29) $$\sum_{\forall (x,y)\in {\text{Kr}}_{3}}k^{x,y}=1$$

(30) $${\text{L}}_{4}^{t,x,y}=\sum_{i=0}^{u-1}\sum_{j=0}^{u-1}{\text{L}}_{3}^{t,x,y}(x+i-a_{i},y+j-a_{j}){\text{Kr}}_{3}$$

(31) $${\text{L}}_{5}^{t,x,y}=\{\begin{array}{cc} 1 & \text{if}\;{\text{L}}_{4}^{t,x,y}>{Τ}_{6}\\ 0 & \text{if}\;{\text{L}}_{4}^{t,x,y}\leq {Τ}_{6} \end{array}$$

Dilation and erosion operations are performed to remove excess noise. The final output (*F_o_*_3_) of the probabilistic edge algorithm is obtained by combining cleaned ${\text{L}}_{3}^{t,x,y}$ and ${\text{L}}_{5}^{t,x,y}$ with an AND operator.

(32) $$F_{o3}^{t,x,y}=\{\begin{array}{ll} 1 & \text{if}\;{\text{L}}_{3}^{t,x,y}={\text{L}}_{5}^{t,x,y}=1\\ 0 & \text{otherwise} \end{array}$$

### Combining Both Outputs of Background Modelling

Since both methods of Sections 2.5 and 2.6 run concurrently, their outputs are independent of each other. In order to make full use of both detections, an OR operator is used so that the detection algorithms can compensate for each other's errors. For each pixel, the output, $F_{o4}^{t,x,y}$ is set to high if any of the method's label is high as shown in Equation (33).

(33) $$F_{o4}^{t,x,y}=\{\begin{array}{ll} 0 & \text{if}\;F_{o2}^{t,x,y}=F_{o3}^{t,x,y}=0\\ 1 & \text{otherwise} \end{array}$$

## 7. Extended Conditional Random Field Shadow & Afterimage Removal

**Figure 6.**
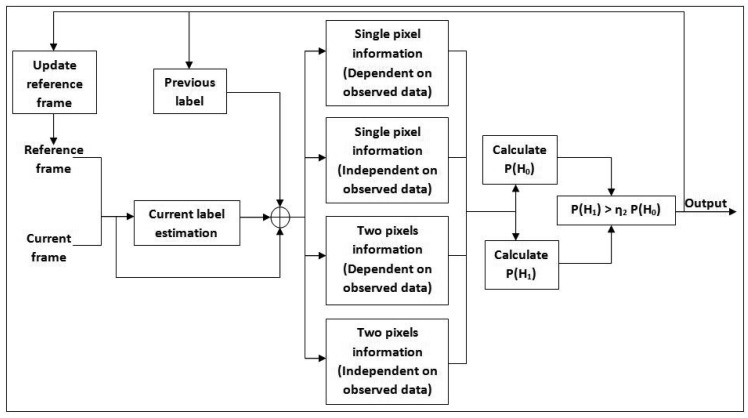
Block diagram of extended CRF shadow & afterimage removal algorithm.

This section describes the suppression of “noise” added during earlier processes by removing the shadow and afterimage. Only pixels recognized as foreground pixel based on $F_{o4}^{t,x,y}$ undergo the removal test. Usually for fast moving objects, the detected foreground is not crisp because of the afterimage noise. In addition, dynamic shadows are also detected as foreground, which creates a double counting problem in people counting systems. The fundamental idea in our approach is the use of conditional random fields as described by Wang [3], which has been improved in [34]. The improved method introduces a fusion of Neyman–Pearson hypothesis testing with extended CRF and a new shadow model. Figure 6 shows a block diagram of the extended CRF shadow and afterimage removal algorithm.

The observation and label for the random field are denoted by **g***^t,x,y^* and **l***^t,x,y^*, respectively. Each label is modelled by *e_k_*, a *k*-dimensional unit vector with its *k^th^* component equal to one. Those vectors are used to segment the label into a real foreground pixel or cast shadow/afterimage pixel. A field can be classified as CRF if it fulfills these two requirements:

1. If the random field, **L** is conditioned on the observed data, **G**.
2. If the random field obeys Markov property:
  $$\begin{array}{ll} P({\text{L}}^{x_{1},y_{1}}|\text{G},{\text{L}}^{x_{2},y_{2}},(x_{2},y_{2})\neq (x_{1},y_{1}))=P({\text{L}}^{x_{1},y_{1}}|\text{G},{\text{L}}^{x_{2},y_{2}}), & (x_{2},y_{2})\in {\text{Kr}}_{4}^{i,j} \end{array}$$
  where ${\text{Kr}}_{4}^{i,j}$ is neighboring sites of pixel at (*i, j*).

Based on the Hammersley–Clifford theorem, a Markov based random field can be shown to be equivalent to a Gibbs random field [3]. In our case, *F_o_*_4_ is taken as the observation field (**G***^t^*), while the random field for the label is denoted as **L***^t^*. Only single potentials *V*^*x*_1_,*y*_1_^ and pairwise potentials *V*^*x*_1_,*y*_1_,*x*_2_,*y*_2_^ will be considered for our algorithm.

(34) $$P({\text{L}}^{t}|{\text{G}}^{t})\propto \{-\sum_{(x_{1},y_{1})\in F}V^{x_{1,}y_{1}}({\text{I}}^{t,x_{1},y_{1}}|{\text{G}}^{t})+\sum_{(x_{2},y_{2})\in {\text{Kr}}_{4}}V^{x_{1},y_{1},x_{2},y_{2}}({\text{I}}^{t,x_{1},y_{1}},{\text{I}}^{t,x_{2},y_{2}}|{\text{G}}^{t})$$

Both the 1-pixel potential and pairwise pixel potential can be broken into two components, either dependent or independent of the observations.

(35) $$V^{x_{1},y_{1}}({\text{I}}^{\text{t},x_{1},y_{1}}|{\text{G}}^{t})=V_{\text{I}}^{x_{1},y_{1}}({\text{I}}^{t,x_{1},y_{1}})+V_{\text{I}|\text{g}}^{x_{1},y_{1}}({\text{I}}^{t,x_{1},y_{1}}|{\text{G}}^{t})$$

(36) $$V^{x_{1},y_{1},x_{2},y_{2}}({\text{I}}^{t,x_{1},y_{1}},{\text{I}}^{t,x_{2},y_{2}}|{\text{g}}^{t})=V_{\text{I}}^{x_{1},y_{1},x_{2},y_{2}}({\text{I}}^{t-1,x_{1},y_{1}},{\text{I}}^{t-1,x_{2},y_{2}})+V_{\text{I}}^{x_{1},y_{1},x_{2},y_{2}}({\text{I}}^{t,x_{1},y_{1}},{\text{I}}^{t,x_{2},y_{2}}+V_{\text{I}|\text{g}}^{x_{1},y_{1},x_{2},y_{2}}({\text{I}}^{t,x_{1},y_{1}},{\text{I}}^{t,x_{2},y_{2}}|{\text{G}}^{t})$$

The independent component of a single pixel potential is modelled as *V***_l_**^*x*_1_,*y*_1_^ (**l***t,x*_1_,*y*_1_) = −*Τ*_7_**l**^*t,x*_1_,*y*_1_^.**l**^*t*−1,*x*_1_,*y*_1_^, while the dependent component of single pixel potential can be further reduced to – ln *P*(**g**^*t,x*_1_,*y*_1_^ |**l***t,x*_1_,*y*_1_). Since we are using Neyman-Pearson hypothesis testing, it will be represented by the likelihood of *H*_0_ and *H*_1_. The probability of detecting a region that is not a shadow, *P*_5_(*H*_0_), is modelled as a Gaussian distribution, which compares the difference between the observation frame *F^t,x,y^*(*R, G, B*) and the reference frame $F_{r}^{t,x,y}(R,G,B)$.

(37) $$\begin{array}{ll} P_{5}(H_{0})\sim NP\left(F_{j}^{t,x,y};F_{r,j}^{t,x,y},\sigma_{5}^{2}\right), & j\in \{R,G,B\} \end{array}$$

On the other hand, the probability of the alternative hypothesis (*P*(*H*_1_)) is obtained by modelling the difference between the observation frame and modified reference frame with Gaussian distribution. Each channel will have its own difference value—in this case, (*d*_1_, *d*_2_, *d*_3_) ∈ *D* for RGB. By using these three difference values, three modified reference frames are established.

(38) $$\left(d_{1}^{t,x,y},d_{2}^{t,x,y},d_{3}^{t,x,y}\right)=\left(\left(F_{R}^{t,x,y}-F_{r,R}^{t,x,y}\right),(F_{G}^{t,x,y}-F_{r,G}^{t,x,y}),\left(F_{B}^{t,x,y}-F_{r,B}^{t,x,y}\right)\right)$$

All three cases are investigated separately, and the minimum output probability is chosen as the null hypothesis probability.

(39) $$\begin{array}{ll} P_{6}(H_{1})\sim \underset{d_{\text{th}}\in D}{\min}NP\left(F_{j}^{t,x,y};d_{i},\sigma_{6}^{2}\right), & j\in \{R,G,B\},d\in D \end{array}$$

The 1-pixel potential only contains temporal information, neglecting the spatial variation. This weakness is overcome by using pairwise pixel potentials where both past and current neighbouring data are taken into consideration for decision making. Let Kr_5_ be the kernel of the neighbourhood with size of *k* × *l* Thus independent component can be modelled as shown in Equations (40) and (41). The first equation represents the spatial relationship between each pixel with its current neighbours while the second equation represents the relationship between each pixel with its past neighbourhood label. All these equations are derived from the assumption that each pixel label will have a higher likelihood to retain its previous label.

(40) $$V_{\text{l}}^{x_{1},y_{1},x_{2},y_{2}}({\text{l}}^{t-1,x_{1},y_{1}},{\text{l}}^{t-1,x_{2},y_{2}})={Τ}_{8}{\text{l}}^{t,x_{1},y_{1}}.{\text{l}}^{t,x2,y_{2}}$$

(41) $$V_{\text{l}}^{x_{1},y_{1},x_{2},y_{2}}({\text{l}}^{t,x_{1},y_{1}},{\text{l}}^{t,x_{2},y_{2}})={Τ}_{9}{\text{l}}^{t-1,x_{1},y_{1}}.{\text{l}}^{t-1,x_{2},y_{2}}$$

The dependent component of the clique potential is the distinguishing factor between a Markov random field (MRF) and a conditional random field. Neighbourhood observation and label relationship will be assumed as zero for MRF approach. Here, we adopt the reduced version of the potential by Wang [3].

(42) $$V_{\text{l}|\text{g}}^{x_{1},y_{1},x_{2},y_{2}}({\text{l}}^{t,x_{1},y_{1}},{\text{l}}^{t,x_{2},y_{2}}|{\text{G}}^{t})={Τ}_{10}\|{\text{g}}^{t,x_{1},y_{1}}-{\text{g}}^{t,x_{2},y_{2}}\|{\text{l}}^{t,x_{1},y_{1}}.{\text{l}}^{t,x_{2},y_{2}}$$

All probability components are put together to get the final label, $L_{6}^{t,x,y}=P({\text{L}}^{t,x,y}|{\text{G}}^{t,x,y})$. A high label is retained from previous subsection if a non-shadow is detected. The pixel will be assigned a low label value if the shadow potential is higher than the non-shadow.

(43) $$L_{6}^{t,x,y}=\{\begin{array}{ll} 1 & \text{if}\;P\;(\text{shadow})\leq P\;(\text{non-shadow})\\ 0 & \text{if}\;P\;(\text{shadow})>P\;(\text{non-shadow}) \end{array}$$

### Maintenance of Reference Image

The reference frame needs to be updated so that it can adapt to changes in surroundings and illumination. Maintenance of the frame is divided into two cases: an illumination change is detected or it is not. When a global illumination change occurs, the current reference frame will no longer be accurate. A new reference frame is initialized by taking the next frame after the illumination change has stabilized.

(44) $$F_{r}^{t+1,x,y}(R,G,B)=F^{t,x,y}(R,G,B)$$

Under constant illumination, the reference frame is updated based on an infinite impulse response filter as in Equation (45) where *Τ*_11_ is a small positive number.

(45) $$F_{r}^{t+1,x,y}(R,G,B)=(1-{Τ}_{11})F_{r}^{t,x,y}(R,G,B)+{Τ}_{11}F^{t,x,y}(R,G,B)$$

## 8. Simulation Results and Discussions

Our algorithm has been tested on various video scenes to prove that accuracy and robustness have been improved over prior algorithms. Its performance has been compared with several existing approaches, including methods by Stauffer and Grimson [2], Li *et al.* [1], Renno *et al.* [7], Wang [3] and Varcheie *et al.* [9]. The parameters used has been tuned to perform as good as possible for that particular video where OpenCV library [37] is used as the basis for coding the methods by Li *et al.* and MoG. Our algorithm was written in C++ using OpenCV library and run on a 2.66 GHz Intel core 2 Duo machine. The processor manages to execute the entire algorithm with the minimum speed of two frames per second for a 960 × 540 frame size. With the help of multicore machines, the algorithm is expected to achieve a speed-up to real-time. The parameters for both MoG and method by Li *et al.* are given in Tables 1 and 2 respectively.

The value of *η* for the Neyman-Pearson hypothesis test is initialized with 0.001 while all variances for Gaussian distribution are initialized as 5. Kernel neighbourhood size can be any odd number, and we obtained acceptable results by implementing 3 × 3 and 5 × 5 kernel sizes. Three evaluation metrics is used to assess the performance of foreground detections, which are total error rate (TER), true positive rate (TPR) and false positive rate (FPR). TER is calculated by taking the ratio between the total number of error pixels and the total number of pixels (TNP). The ground truth image has been processed manually, which is the reference for identifying the error pixels. The total number of errors is a combination of the false positive (*f_p_*) and false negative (*f_n_*) pixels. False positive is an error where the pixel is detected as foreground, but it is actually not. False negative occurs due to misdetection where the foreground pixel is recognized as a background. Total error rate is calculated as follows:

(46) $$\text{TER}=\frac{f_{p}+f_{n}}{\text{TNP}}\times 100\%$$

TPR and FPR are used to indicate the algorithm performance in terms of correct and wrong detection of foreground pixels respectively. Both metrics gives an output in the range of [0, 1]. A TPR of 1 signifies the best true detection where no wrong background pixels are detected. On the other hand, a FPR of 0 indicates that no wrong foreground pixels are detected. Therefore, a good foreground detector should have high TPR and low FPR values. Both metrics are calculated as follows where *t_p_* and *t_n_* are true positive and true negative respectively.

(47) $$\text{TPR}=\frac{t_{p}}{t_{p}+f_{n}}$$

(48) $$\text{FPR}=\frac{f_{p}}{f_{p}+t_{n}}$$

The analysis is separated into six categories, where we refer our algorithm as Zulkifley *et al.* The first and second videos are used to test the overall performance of the algorithms while the third, fourth and fifth videos are used to verify specific performance improvement of the subsystems. The role of the third video is to point out the advantage of using the masks in grey world algorithm compared to Renno *et al.* [7] algorithm. The purpose of the fourth video is to demonstrate the advantage of fusing probabilistic edge algorithm compared to using the Li *et al.* [1] algorithm alone. The fifth video will test the performance difference between our shadow model and the Wang [3] shadow model. The last test compares our algorithm performance with respect to the state-of-the-art method, which is based on the algorithm by Varcheie *et al.* [9]. Videos 1, 4 and 5 will demonstrate performance improvement of using our method compared to the method by Varcheie *et al.*

**Figure 7.**
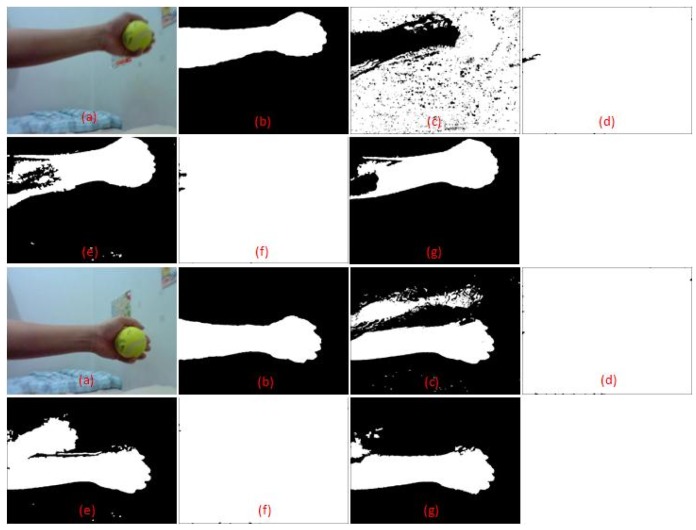
Simulation results: (**a**) Input (**b**) Ground truth (**c**) MoG (**d**) Method: Li *et al.*; (**e**) Method: Renno *et al.*; (**f**) Method: Yang Wang (**g**) Method: Zulkifley *et al.*

**Figure 8.**
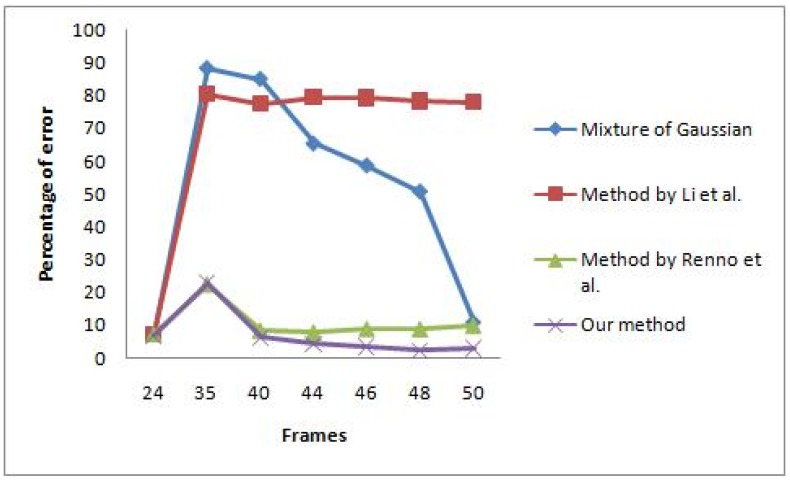
Rate of response of the algorithms under sudden illumination change.

The first video contains a scene where global illumination has changed from a bluish to a more reddish illumination because of a lighting change. Some frames of the scene are shown in Figure 7, which is taken directly after sudden illumination change occurred. The learning rate for both MoG and colour co-occurrence methods is set to normal, which has been tuned for good performance in sudden and gradual illumination change. The video also contains some afterimage noise because of fast movement of the hand. The performance comparison between the algorithms is shown in Figure 8. Only the methods by Zulkifley *et al.* and Renno *et al.* [7] managed to maintain an acceptable error rate after a sudden illumination change, which occurs at frame number 35. After 15 frames have lapsed, the MoG method manages to retrieve an acceptable error rate while the Wang [3] and Li *et al.* [1] algorithms still fail to obtain an acceptable error rate. We selected an acceptable error rate below 10% as most of the papers [1,3,7] reported their error rate as less than 10%. Table 3 shows the total error rate which clearly indicates that our algorithm performed better than all the others. Note that every algorithm includes small component removal.

The result supports our earlier argument that it is hard to choose a single background learning rate to accommodate both sudden and gradual illumination changes. Our method lets colour constancy react to sudden illumination changes while the background learning rate is set to handle gradual change. Our 2-stage masked grey world managed to stabilize the input image, especially during the abrupt change in the illumination. This allows our background modelling to be more accurate as not much difference is detected due to good normalized input image. We also found that the methods by Li *et al.* and Wang had a higher TPR compared to us, which indicate better true positive detection. However, their FPR values are also high while ours is only 0.035. A high FPR value signifies that many background pixels are detected as foreground. This explains the reason why our TER is the lowest, which is supported by good TPR and FPR values. Therefore, our algorithm performs the best compared to the others, especially during the illumination change scene.

**Figure 9.**
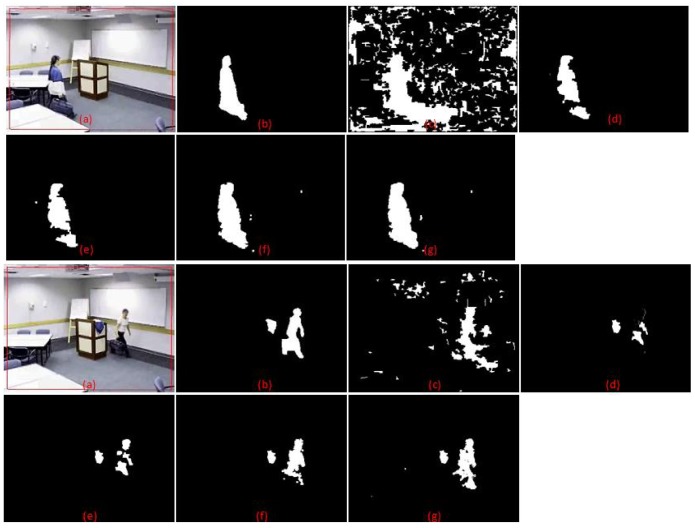
Simulation results: (**a**) Input (**b**) Ground truth (**c**) MoG (**d**) Method: Li *et al.*; (**e**) Method: Renno *et al.*; (**f**) Method: Yang Wang; (**g**) Method: Zulkifley *et al.*

Samples of the second video scene are shown in Figure 9. It contains a complex situation in which the moving object appears similar to the background colour. The moving person took off her jacket and left it on the rostrum. She then walked in front of a white board in a white t-shirt. This poses a challenge for any algorithm that is dependent on colour information alone where the information will be quite similar. There is also an afterimage and shadow effect, which contributes to additional noise. Table 4 shows that the method by Zulkifley *et al.* has the lowest TER, which is just 1.49%, while MoG performs the worst at 6.79% error rate. The result also shows that we have the highest TPR value at 0.844. This proves that we managed to increase foreground detection in challenging situations, especially for the colour similarity issue. Our FPR is 0.011, not the lowest but still a good value. This small increment in false detection rate is a worthy trade-off for higher true detection. Note that MoG has the highest error rate since it only utilized colour information for detection where the shirt data has been recognized as the background data.

**Figure 10.**
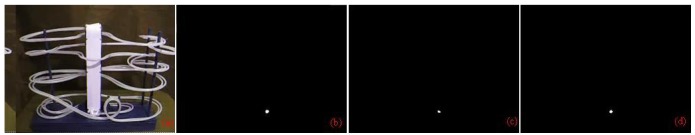
Simulation results for the grey world algorithm. (**a**) Input (**b**) Ground truth (**c**) Method: Renno *et al.* (**d**) Zulkifley *et al.*

**Figure 11.**
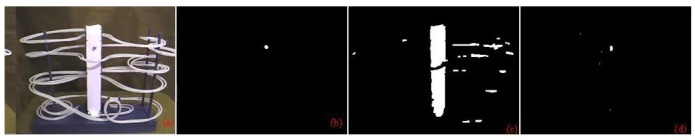
Simulation results for the grey world algorithm. (**a**) Input (**b**) Ground truth (**c**) Method: Renno *et al.* (**d**) Zulkifley *et al.*

For the third video, the foreground object is a marble with variable speeds, as shown in Figures 10 and 11. Initially, the marble moves slowly and then after a sudden illumination change occurred, the marble rolls faster, creating some afterimage noise. Accuracy comparison is calculated between the methods by Zulkifley *et al.* and Renno *et al.* The result shows that our algorithm manages to react better to sudden illumination change as shown in Table 5 by introducing a mask in the grey world algorithm. Our method manages to reduce the error rate of foreground detection from 5.055% to 0.047%. In this video, misdetection is a critical issue since the object is very small. Our TPR is worse than the method by Renno *et al.* but our FPR value is better. This signifies that we managed to reduce false detection even for such a small object due to our good shadow and afterimage removal. Another reason why our algorithm produced better detection is due to foreground information subtraction while calculating grey constants. Therefore, more accurate normalization constants are obtained to lessen the effect of illumination change.

**Figure 12.**
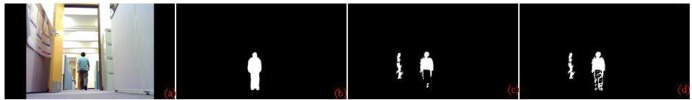
Simulation results for the colour similarity case. (**a**) Input (**b**) Ground truth (**c**) Method: Li *et al*; (**d**) Method: Zulkifley *et al.*

**Figure 13.**
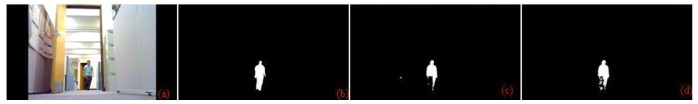
Simulation results for the colour similarity case. (**a**) Input (**b**) Ground truth (**c**) Method: Li *et al.*; (**d**) Method: Zulkifley *et al.*

The fourth video is chosen to show the advantage of fusing edge and intensity information for background modelling. The foreground object in the test video is a moving human with dark grey trousers. He walked in front of a black colour background as shown in Figures 12 and 13. The performance of our algorithm is compared with the method by Li *et al.*, which depends on colour values alone for background modelling. As shown in Table 6, the average error rate is reduced from 1.039% to 0.880%. Although the error reduction appears small quantitatively, in terms of qualitative analysis our algorithm managed to get more accurate outer appearance of the moving object. This is very important, especially for people counting systems in a complex scene where the bounding box size is used to determine the number of people. Our algorithm manages to increase the true detection as shown by a higher TPR value compared to the method by Li *et al.* Although our FPR has a higher value of 0.004 compared to 0.003 from the method by Li *et al.*, this small increment can be neglected as better detection of true positive is obtained.

**Figure 14.**
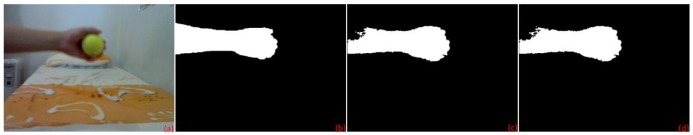
Simulation results for the shadow modelling comparison. (**a**) Input (**b**) Ground truth (**c**) Method: Wang (**d**) Method: Zulkifley *et al.*

**Figure 15.**
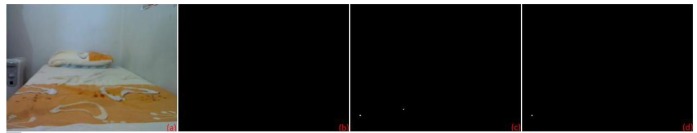
Simulation results for the shadow modelling comparison. (**a**) Input (**b**) Ground truth (**c**) Method: Wang (**d**) Method: Zulkifley *et al.*

The fifth video scenes shown in Figures 14 and 15 are used to compare the performance of the shadow model between Wang's [3] method and our algorithm. The scenes contain a moving hand where shadows are formed at the bottom of the frame. Figure 15 is quite a challenging scene since the shadow can still be seen at the bottom of the frame even though the hand is already out of the scene. Table 7 shows the error analysis of the scenes. There is no significant performance difference as algorithm 2(d) performs slightly better with 0.5486% error rate compared to 0.5538% for Wang's algorithm. Our TPR value is lower by 0.001 while our FPR value is higher by 0.0001. We can conclude that additional information from the past neighbourhoods did not improve shadow removal capability for this particular video.

**Figure 16.**
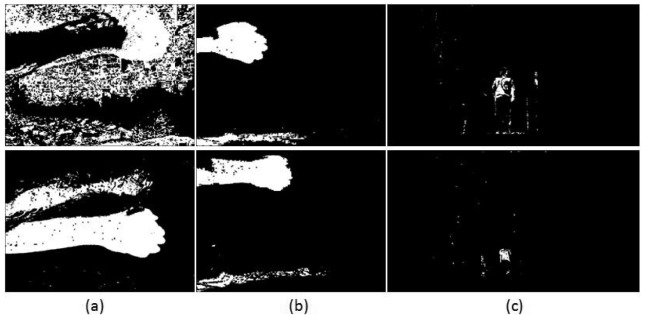
Samples of simulation results for the method by Varcheie *et al.* (**a**) Video 1 (**b**) Video 4 (**c**) Video 5.

The last results are meant to compare our algorithm performance with the state-of-the-art method. Method by Varcheie *et al.* was selected as the benchmark. This method is a derivative of mixture of Gaussian approach where selective updating is used. The frame is divided into variable size of boxes. The histogram and variance of each box are generated where the background model is based on the first frame data. It will be updated with the current information if any boxes are deemed to be the background. If the certain threshold of histogram and variance difference are met, the boxes are considered as the foreground region. This particular region is then updated by using the mixture of Gaussian method. This approach will not increase the detection of foreground pixels since the foundation is still MoG, yet it will reduce false detection since it filtered out any small region noise that has size of less than 4 × 3 pixels. For all three tested video, our TER values are less than the method by Varcheie *et al.* as shown in Table 8. Same conclusion can be made to our TPR values, which are higher for the tested videos. It shows that in the presence of sudden illumination change, shadow noise and colour similarity between foreground object and background, our algorithm performed better than the method by Varcheie *et al.* For the FPR values, Varcheie *et al.*'s method produced a better result for video 4 only compared to our algorithm. This is because our algorithm detected more foreground pixels, especially in challenging situations. Thus, more false positive is generated but the number is kept at the minimum through our shadow removal process. Basically, the method by Varcheie *et al.* will suffer the same problem as MoG but with reduced false detection. Some output samples of the algorithm by Varcheie *et al.* can be found in Figure 16.

## 9. Conclusions

We have presented a novel approach to enhance the robustness and accuracy of foreground detection. The integrated algorithm has been tested and proven to be robust to (1) colour similarity between background and foreground objects; (2) shadows and afterimages noise; and (3) sudden and gradual illumination changes. The main novelties of the algorithm are the introduction of 2-stage mask for grey world algorithm, probabilistic approach to edge-based background modelling and extended CRF shadows removal. Our algorithm is suitable to be applied in systems that require robust foreground detection such as face recognition, people counting, traffic monitoring and robotic vision. This work can be further improved in the future by using faster processor such as Field Programmable Gate Array (FPGA) [38]. Moreover, a more integrated modelling can be used to reduce the redundancy in some of the detections.

## Figures and Tables

**Table 1.** Parameters used for the mixture of Gaussian method.

**Parameter**	**Value**
Background test threshold	0.7
Standard deviation threshold for Gaussian distribution	2.5
Window size	200
Number of Gaussian distribution	5
Initial weight of each Gaussian	0.05
Initial variance of each Gaussian	30
Minimum number of pixels (for clustering)	15

**Table 2.** Parameters used for colour co-occurrence background modelling.

**Parameter**	**Value**
Update parameter for background reference	0.7
Learning constant	0.005
Number of colour vectors (normal background model)	15
Number of colour vectors (after update)	25
Number of colour co-occurrence vectors (normal background model)	25
Number of colour co-occurrence vectors (after update)	40
Minimum number of pixels (for clustering)	15

**Table 3.** Performance comparison between the methods for the first video.

**Method**	**TER**	**TPR**	**FPR**
Mixture of Gaussian	54.14%	0.805	0.635
Li *et al.* algorithm	78.43%	0.998	0.999
Renno *et al.* algorithm	8.71%	0.950	0.097
Wang algorithm	78.47%	0.998	0.999
Zulkifley *et al.* algorithm	3.87%	0.950	0.035

**Table 4.** Performance comparison between methods for the second video.

**Method**	**TER**	**TPR**	**FPR**
Mixture of Gaussian	6.79%	0.726	0.062
Li *et al.* algorithm	1.71%	0.548	0.005
Renno *et al.* algorithm	1.67%	0.588	0.006
Wang algorithm	1.58%	0.678	0.008
Zulkifley *et al.* algorithm	1.49%	0.844	0.011

**Table 5.** Performance comparison between Renno *et al.* method and Zulkifley *et al.* algorithm.

**Method**	**TER**	**TPR**	**FPR**
Renno *et al.*	5.055%	0.891	0.053
Zulkifley *et al.*	0.047%	0.699	0

**Table 6.** Performance comparison between Li *et al.* method and Zulkifley *et al.* algorithm.

**Method**	**TER**	**TPR**	**FPR**
Li *et al.*	1.039%	0.528	0.003
Zulkifley *et al.*	0.880%	0.655	0.004

**Table 7.** Performance comparison between Wang's method and Zulkifley *et al.* algorithm.

**Method**	**TER**	**TPR**	**FPR**
Wang	0.5538%	0.535	0.0042
Zulkifley *et al.*	0.5486%	0.534	0.0041

**Table 8.** Performance comparison between the methods by Varcheie *et al.* and Zulkifley *et al.*

**Method**	**Video**	**TER**	**TPR**	**FPR**
Varcheie *et al.*	Video 1	35.36%	0.792	0.391
Zulkifley *et al.*	Video 1	3.87%	0.950	0.035

Varcheie *et al.*	Video 4	1.318%	0.296	0.002
Zulkifley *et al.*	Video 4	0.880%	0.655	0.004

Varcheie *et al.*	Video 5	3.815%	0.462	0.027
Zulkifley *et al.*	Video 5	0.549%	0.534	0.004
